# Staining Room Floors

**Published:** 1885-08

**Authors:** 


### Staining Room Floors.

The practice of staing board floors a darker tint than their original color, or to represent woods greater cost, is now almost universal, and is the first step toward that thing of beauty, a high-art drawing-room. A floor stained to represent dark old oak is preferred by many. The mixture for accomplishing this is sold at all paint shops, and comes in grades 1, 2, 3, 4, varying light to dark. Light polished floors are much preferred at present, although a great deal depends upon the condition of the boards. If the boards are smooth and fine-grained, a satinwood or pitch-pine stain and polish is preferred ; but if the floor is old or rough it is folly to attempt any stain except that of dark oak or dark mahogany. Pour the liquid into a saucer, dip the brush in, saturate thoroughly, rub evenly over the wood and dry instantly with a soft cloth. To ensure success this must be done quickly and evenly. For the ultra-fashionable floor, which is a pale shade of oak, sized and varnished, buy the desired amount of raw sienna powder, mixed with water, and rub into the boards as directed above. For mahogany staining make a mixture containing half a pound of madder, two ounces of logwood chips boiled in a gallon of water ; brush this over the wood while hot. When dry go over this with a solution of pearl-ash, two drams to one quart of water, size and polish. If a redder shade is required it can be produced by smearing the surface with a strong solution of permanganate potash, which is left on for a longer or shorter time, according to the shade required ; in most cases five minutes will be enough. The wood is then carefully washed, dried and polished in the ordinary way. A good cheap oak stain is made of equal parts of American potash and pearl-ash, two ounces of each to a quart of water. As American potash is a solvent, care should be taken to keep it from the hands, and an old brush should be used as it is no good afterward.—*Exchange.*

